# OL-FS13 Alleviates Cerebral Ischemia-reperfusion Injury by Inhibiting miR-21-3p Expression

**DOI:** 10.2174/1570159X21666230502111013

**Published:** 2023-09-25

**Authors:** Naixin Liu, Yan Fan, Yilin Li, Yingxuan Zhang, Jiayi Li, Yinglei Wang, Zhuo Wang, Yixiang Liu, Yuansheng Li, Zijian Kang, Ying Peng, Zeqiong Ru, Meifeng Yang, Chengan Feng, Ying Wang, Xinwang Yang

**Affiliations:** 1 Department of Anatomy and Histology & Embryology, Faculty of Basic Medical Science, Kunming Medical University, Kunming, 650500, Yunnan, China;; 2 Key Laboratory of Chemistry in Ethnic Medicinal Resources & Key Laboratory of Natural Products Synthetic Biology of Ethnic Medicinal Endophytes, School of Ethnic Medicine, Yunnan Minzu University, State Ethnic Affairs Commission & Ministry of Education, Kunming, Yunnan, 650504, China

**Keywords:** Cerebral ischemia-reperfusion, microRNA, CAMKK2/AMPK pathway, peptide, neuroprotective, oxidative stress

## Abstract

**Background:**

OL-FS13, a neuroprotective peptide derived from *Odorrana livida*, can alleviate cerebral ischemia-reperfusion (CI/R) injury, although the specific underlying mechanism remains to be further explored.

**Objective:**

The effect of miR-21-3p on the neural-protective effects of OL-FS13 was examined.

**Methods:**

In this study, the multiple genome sequencing analysis, double luciferase experiment, RT-qPCR, and Western blotting were used to explore the mechanism of OL-FS13.

**Results:**

Showed that over-expression of miR-21-3p against the protective effects of OL-FS13 on oxygen-glucose deprivation/re-oxygenation (OGD/R)-damaged pheochromocytoma (PC12) cells and in CI/R-injured rats. miR-21-3p was then found to target calcium/calmodulin-dependent protein kinase 2 (CAMKK2), and its overexpression inhibited the expression of CAMKK2 and phosphorylation of its downstream adenosine 5’-monophosphate (AMP)-activated protein kinase (AMPK), thereby inhibiting the therapeutic effects of OL-FS13 on OGD/R and CI/R. Inhibition of CAMKK2 also antagonized up-regulated of nuclear factor erythroid 2-related factor 2 (Nrf-2) by OL-FS13, thereby abolishing the antioxidant activity of the peptide.

**Conclusion:**

Our results showed that OL-FS13 alleviated OGD/R and CI/R by inhibiting miR-21-3p to activate the CAMKK2/AMPK/Nrf-2 axis.

## 1 INTRODUCTION

Globally, more than 10 million people suffer a stroke yearly, with associated complications such as reduced motor function and neurobehavioral changes [1, 2]. Stroke is broadly categorized as ischemic or hemorrhagic, with ischemic stroke (IS) being the most common (approximately 87%) [3-5]. IS is usually caused by arterial thrombosis, which can lead to cerebral infarction and neurological deficits [6, 7]. While early reperfusion is a recognized treatment for IS, cerebral ischemia/reperfusion (CI/R or I/R) does not reverse the pathological changes caused by ischemia, such as an injury due to oxygen-glucose imbalance, adenosine triphosphate (ATP) depletion, and oxidative stress. It may even aggravate neuronal death [8-10]. Therefore, intervention strategies that can reduce CI/R damage are essential.

Brain tissue affected in CI/R is divided into two regions, *i.e*., the ischemic core and the surrounding penumbra [9, 11]. Core neurons die rapidly due to necrosis, whereas neurons in the penumbra are less ischemic and can potentially survive, despite functional inhibition, making the penumbra a target for therapeutic intervention [12, 13]. The pathological process of CI/R injury is complex and includes oxidative stress, excitotoxicity, intracellular calcium overload, inflammation, and apoptosis [14-16]. Therefore, in developing new and effective drug targets for CI/R, the combination with the pathophysiological process is necessary, which is also the core of the research to conquer CI/R [17].

In recent years, microRNA (miRNA) gene expression regulators have become an important focus in disease research [18, 19]. As a family of small noncoding RNAs, miRNAs (by binding to the 3’-untranslated region (UTR) of downstream target messenger RNA (mRNA)) can affect various pathophysiological processes of diseases [20, 21]. Evidence suggests that miRNAs may be key in treating neurological diseases [22-24]. However, further research requires further research on the relationship between pathogenesis, treatment, and miRNA expression in CI/R.

We previously reported a neural-protective peptide (OL-FS13) from *Odorrana livida* (*O. livida*), which exhibited an inhibitory effect on oxygen-glucose deprivation/reoxygenation (OGD/R)-induced oxidative stress and endoplasmic reticulum stress injury by activating the nuclear factor erythroid 2-related factor 2 (Nrf-2) and reducing neuronal cell apoptosis [25].

Here, we found that miR-21-3p and calcium/calmodulin-dependent protein kinase 2 (CAMKK2) were significantly altered in CI/R rats treated with OL-FS13. Online prediction further showed that miR-21-3p and CAMKK2 might have a targeting relationship. Thus, to determine whether miR-21-3p/ CAMKK2 is related to the effects of OL-FS13 in CI/R treatment, we evaluated the effect of the miR-21-3p/CAMKK2/ adenosine 5’-monophosphate (AMP)-activated protein kinase (AMPK) axis on OL-FS13 treatment. This research could provide more evidence for the application of OL-FS13 in CI/R drug therapy.

## 2 MATERIALS AND METHODS

### 2.1 Materials and Animals

OL-FS13 (FSLLLTWWRRRVC, HPLC 98%) was synthesized and provided by Bioyeargene Biotechnology Co., Ltd. (Hubei, China) [25].

STO-609 and ML385 were obtained from MedChemExpress (New Jersey, USA). Reagents related to cell culture were all obtained from BI (Israel).

A cell viability assay kit (MTS) was provided by Promega (Madison, USA). The 2, 3, and 5-triphenyte-trazoliumchloride (TTC) was from Sigma (MO, USA). The staining kits, protein extraction kits, and BCA kits were obtained from Solarbio (Beijing, China). Primary antibodies Bcl-2, lamin B1, β-actin, Bax, Nrf2, caspase 3, cleaved caspase 3, CAMKK2, AMPK, and pAMPK (1:1000, anti-rabbit), as well as secondary antibodies (1:5000, anti-rabbit), were purchased from Proteintech (Hubei, China). The RNA extraction kit was from Tiangen Biotech (Beijing, China). The miR-21-3p, U6, CAMKK2, and actin primers were synthesized and obtained from GeneCopoeia (Guangzhou, China). The cDNA reverse transcription kit and qPCR amplification kit were obtained from GeneCopoeia (Guangzhou, China). The miR-21-3p mimic, miR-21-3p mimic NC, miR-21-3p inhibitor, miR-21-3p inhibitor NC, miR-21-3p agomiR, and cell transfection kits were all from Ribobio Biotechnology Co., Ltd. (Guangzhou, China).

Pheochromocytoma (PC12) cells (KCB 93033YJ) were obtained from the Kunming Institute of Zoology (Kunming, China).

Adult male Sprague-Dawley (SD) rats (260-280 g, n = 60) were obtained from SPF (Beijing) Biotechnology Co., Ltd. (Beijing, China).

### 2.2 Construction of Animal Models

CI/R surgery in rats was performed as reported previously [26, 27]. All rats were observed for 24 h after the following intervention measures: (1) OL-FS13 (10 μg/kg) was administered through the tail vein after reperfusion; (2) I/R and control group was given ddH_2_O as placebo; (3) agomiR (5 nmol) was injected through the tail vein before operation; (4) STO-609 (15 mg/kg) and ML385 (25 mg/kg) were administered (intraperitoneal injection) once a day for three days before surgery.

### 2.3 mRNA and microRNA Sequencing

RNA in the cerebral cortex of rats was obtained to detect and compare the types and quantities of mRNA and miRNA in each group (control, I/R, OL-FS13 groups, n = 3) [19]. In brief, the cerebral cortices were removed and stored in RNAlater (100 mg/mL), then sent to Beijing Guoke Biotechnology Co., Ltd. (Beijing, China) for mRNA and miRNA transcriptome detection and analysis.

### 2.4 Dual-luciferase Reporter Assay

TargetScan (http://www.targetscan.org/vert_71/) and miRDB (http://www.mirdb.org/) were used for target prediction of miR-21-3p [28, 29]. Dual luciferase assays of miR-21-3p and CAMKK2 were performed and reported by UPTBIO (Hunan, China).

### 2.5 Cell Culture, OGD/R Construction, and Viability Assay

Cell culture and OGD/R construction were performed [25, 30]. The OGD environment was established using glucose-free DMEM and 95% N_2_. Cells were re-oxygenated and provided glucose after 4 h of ischemia and hypoxia (the control group was cultured under general conditions) [31-33]. According to the different groups, the following interventions were performed: (1) OL-FS13 (100 pM) was administered after re-oxygenation/glucose addition; (2) OGD/R and control groups were given phosphate-buffered saline (PBS); (3) Transfection experiments were performed as the manufacturer’s instruction. Following transfection, OGD/R was performed after 24 h of general culture; (4) STO-609 (3 μM) and ML385 (5 μM) were administered 24 h before OGD/R. Cell viability was detected by the MTS kit.

### 2.6 RT-qPCR

Total RNA and miRNA in cells and tissues were obtained using RNA extraction kits per the manufacturer’s instructions [26, 34]. The obtained RNAs (1.5 μg) were reverse transcribed into cDNA using the related kits. The CT numbers of mRNA and miRNA in each group were obtained using a PCR detector (Life Technologies, Thermo, USA) [35].

### 2.7 Western Blotting

Total, cytosolic, and nuclear protein in cells and tissues were obtained using protein extraction kits according to the provided instructions [25, 36]. Protein concentration was detected using a BCA kit. Protein (30 μg) was then separated by SDS-PAGE and electro-transferred to PVDF membranes. After blocking with skim milk, the membranes were incubated with primary antibodies overnight, then with secondary antibodies at room temperature. The protein expression intensity was then calculated.

### 2.8 Evaluation of Nerve Function and Infarct Volume

The modified neurological severity score (mNSS) was used to evaluate the degree of neurological injury in rats, and infarct volume was observed by TTC staining [37]. The whole brains of rats were obtained and frozen for coronal sectioning (six sections) [38]. The sections reacted with 1% TTC solution at 37°C for 15 minutes. Infarct volume = 100% × infarct area ÷ total area (calculated by Image J) [39].

### 2.9 Histological Examination

H&E and Nissl staining were used to study CI/R-induced injury histologically [40]. Whole brains were fixed in 4% paraformaldehyde for 48 h, then embedded in paraffin, sectioned into 5-8 μm sections, and stained using H&E and Nissl staining kits. Xylene was first used to remove the paraffin from tissue sections and then hydrated by gradient ethanol (ethanol concentration from 100% to 75%) and staining by staining kits [41] in briefly, H&E staining: hematoxylin (2 min) - Differentiation fluid (30 s)-eosin (1 min); Nissl staining: tar purple (1 h) - differentiation liquid (2 min). The stained sections were then handled by gradient ethanol (ethanol concentration from 75% to 100%) and xylene before sealing with neutral gum for preservation and observation.

A vertical microscope (Zeiss, Germany) was used to observe the results and obtain images.

### 2.10 Statistical Methods

Data are presented as mean ± SEM. GraphPad Prism v7 was used to analyze the experimental results and draw pictures. Comparisons between two groups were performed using a *t-*test, and comparisons between more than two groups were performed by a one-way analysis of variance (ANOVA) test. Here, *P* > 0.05 was considered as not statistically different. All experiments were repeated three times independently.

## 3 RESULTS

### 3.1 OL-FS13 May Play a Neuroprotective Role *via* miR-21-3p/CAMKK2/AMPK Axis

#### 3.1.1 Multi-omics Sequencing Analysis

**Fig. (1):**
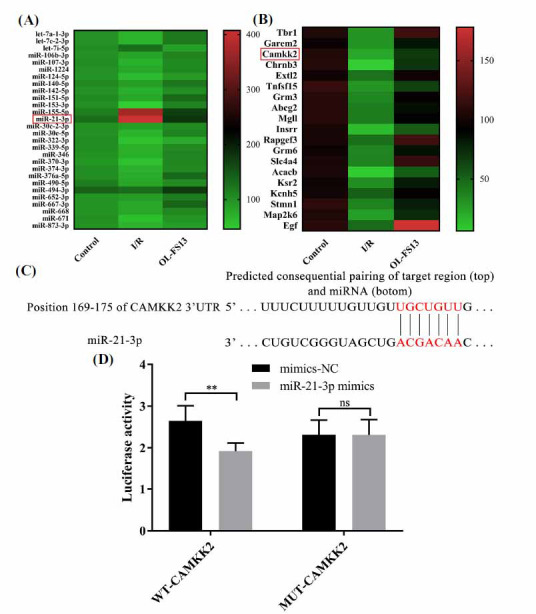
RNA profile and targeting relationship in rat cerebral cortex after I/R. (**A** and **B**) Heat map of differentially expressed miRNA (**A**) and mRNA (**B**) associated with neuroprotection of OL-FS13.The miRNA and mRNA which most strongly associated with neuroprotection were circled by red rectangular boxes; (**C** and **D**). Dual luciferase reporter assay showed that miR-21-3p targeted CAMKK2. **P<0.01, ^ns^*P* ≥ 0.05.

To prove the influence of miRNAs in the function of OL-FS13, we performed multi-omics sequencing analysis (miRNA and mRNA). As Fig. (**1A**) showed, miR-21-3p was the most significantly down-regulated gene (OL-FS13 *vs*. I/R). Based on a comparative analysis of the mRNA targeting prediction and sequencing results (Fig. **1B**), CAMKK2 was one of the targets of miR-21-3p. Thus, we then evaluated the relationship of miR-21-3p/CAMKK2.

#### 3.1.2 miR-21-3p Targeted CAMKK2

Dual luciferase reporter assay can detect the targeting relationship of miRNAs-mRNAs [42, 43]. As Fig. (**1C**) showed, the miR-21-3p mimic inhibited the expression of wild-type CAMKK2 (WT-CAMKK2) but did not affect the expression of CAMKK2 with a 3’UTR mutation (MUT-CAMKK2), indicating that miR-21-3p targeted CAMKK2.

#### 3.1.3 Expression of miR-21-3p/CAMKK2/AMPK Under CI/R and OGD/R

**Fig. (2):**
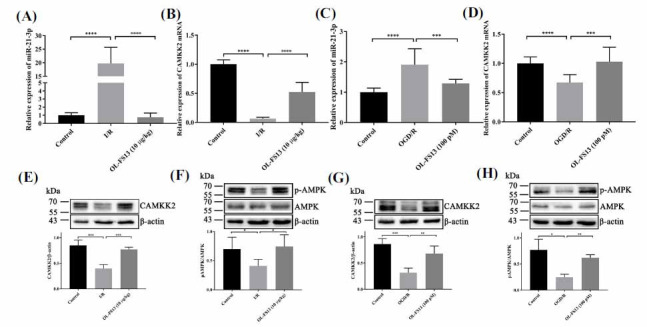
Expression of miR-21-3p, CAMKK2, and AMPK *in vivo* and *in vitro* after OL-FS13 intervention. (**A**-**D**). RT-qPCR results for the expression level of miR-21-3p (**A** and **C**) and CAMKK2 mRNA (**B** and **D**) in the cerebral cortex of I/R rats after OL-FS13 intervention and in PC12 cells after OGD/R; (**E-H**). Expression level of CAMKK2, pAMPK/AMPK *in vitro* and *in vivo*. ****P < 0.0001, ***P < 0.001, **P < 0.01, *P<0.05.

CAMKK2 is a calcium regulatory protein, in which AMPK is the most characterized substrate [44, 45]. In I/R, the CAMKK2/AMPK signaling pathway helps maintain energy balance and resist aberrant ATP consumption [44, 46]. Here, miR-21-3p/CAMKK2/AMPK expression was evaluated in CI/R rats and OGD/R-injured PC12 cells to determine its effect on OL-FS13 neuroprotection. As shown in Figs. (**2A** and **2B**), miR-21-3p expression was up-regulated, and CAMKK2 mRNA expression was down-regulated in the CI/R rats (*vs*. control). Similarly, miR-21-3p was increased (Fig. **2C**), and CAMKK2 mRNA was decreased in the PC12 cells (Fig. **2D**). Furthermore, CAMKK2/AMPK expression was significantly down-regulated in rats and PC12 cells (Figs. **2E-H**). However, these changes were all reversed by OL-FS13 intervention, suggesting the neuroprotective effects of OL-FS13 may be related to the miR-21-3p/CAMKK2/AMPK axis.

### 3.2 Over-expression of miR-21-3p Inhibited Neuroprotective Activity of OL-FS13 *in vitro*

#### 3.2.1 Effect of miR-21-3p Mimic and Inhibitor on miR-21-3p/CAMKK2 Expression in PC12

The expression of miR-21-3p and CAMKK2 mRNA was determined by RT-qPCR assay. As Figs. (**S1A** and **S1B**) showed, the mimic increased the expression of miR-21-3p and decreased that of CAMKK2 mRNA. Correspondingly, the inhibitor decreased the levels of miR-21-3p and increased that of CAMKK2 mRNA. Western blotting also found the mimic and inhibitor decreased and increased CAMKK2 expression, respectively. In addition, neither vector (mimic-NC and inhibitor-NC) affected CAMKK2 expression (Fig. **S1C**, *P* ≥ 0.05 *vs*. Control). These results suggest that mimic and inhibitor of miR-21-3p regulate miR-21-3p/CAMKK2 levels in PC12 cells.

#### 3.2.2 Overexpression of miR-21-3p Decreased Effects of OL-FS13 on OGD/R

**Fig. (3):**
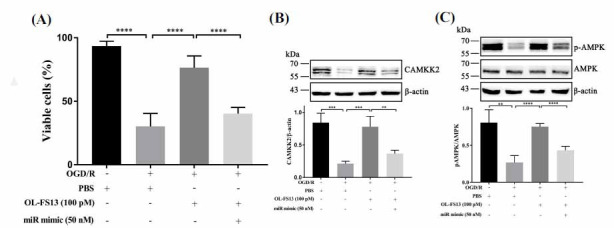
Overexpression of miR-21-3p inhibited the protective effect of OL-FS13 on PC12 cells. (**A**) Viability of PC12 cells. The treatment of OL-FS13 alleviated OGD/R-induced cell injury and was inhibited by overexpression of miR-21-3p; (**B** and **C**). The expression level of CAMKK2 and pAMPK/AMPK *in vitro* and *in vivo*. ****P < 0.0001, ***P < 0.001, **P < 0.01.

Cell viability (after OGD/R) was performed to evaluate after miR-21-3p overexpression [47]. Cell viability was only 30.33% ± 11.33% in the OGD/R group (Fig. **3A**, control group as 100%) but increased to 76.48% ± 9.67% under OL-FS13 intervention. The effect of OL-FS13 on cell viability was significantly decreased (40.39% ± 1.29% *vs*. control) under mimic interference. The results showed that miR-21-3p mimic inhibits the ameliorating effects of OL-FS13 after OGD/R.

#### 3.2.3 miR-21-3p Mimic Inhibited Regulatory Effects of OL-FS13 on the CAMKK2/AMPK Signaling Pathway in OGD/R

CAMKK2/AMPK pathway expression under miR-21-3p mimic treatment was detected to verify whether OL-FS13 mediates the CAMKK2/AMPK signaling pathway through miR-21-3p. As shown in Figs. (**3B** and **3C**), CAMKK2/ AMPK pathway expression was significantly inhibited by OGD/R (Control *vs*. OGD/R, *P* < 0.001) but improved under OL-FS13 administration (OGD/R *vs*. OL-FS13, *P* < 0.001). In addition, CAMKK2/AMPK pathway expression in the mimic group (treated with OL-FS13) was significantly decreased (OL-FS13 *vs*. mimic, *P* < 0.01). Thus, OL-FS13 appears to activate the CAMKK2/AMPK signaling pathway by inhibiting miR-21-3p, which may be the reason for its neuroprotective ability.

### 3.3 Overexpression of miR-21-3p Inhibited the Neural-protective Activity of OL-FS13 on Rats

#### 3.3.1 Effect of miR-21-3p agomiR in the Cerebral Cortex of Rats

As shown in Figs. (**S2A** and **S2B**), agomiR increased miR-21-3p expression and decreased CAMKK2 mRNA expression in the cerebral cortex of rats [48]. Furthermore, the agomiR also inhibited the expression of CAMKK2 in the brain tissue (Fig. **S2C**). Thus, these results suggest that agomiR administration can regulate miR-21-3p/CAMKK2 in the cerebral cortex of rats.

#### 3.3.2 miR-21-3p agomiR Inhibited Effects of OL-FS13 on Infarct Volume and Neurological Function in CI/R Rats

**Fig. (4):**
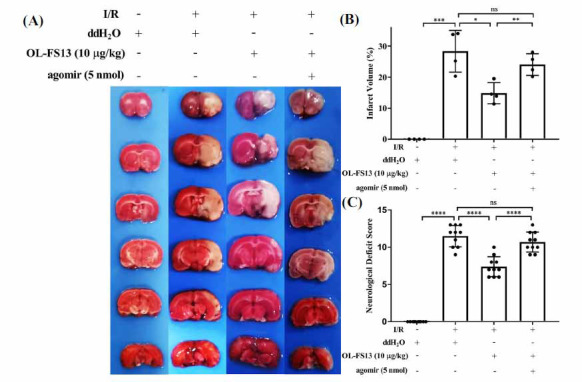
The effect of miR-21-3p overexpression inhibited the improvement of OL-FS13 on cerebral infarct volume and neurological function in I/R rats. (**A** and **B**) TTC staining; (**C**) Score of neurological function injury in rats. ****P < 0.0001, ***P < 0.001, **P < 0.01, *P < 0.05, ^ns^*P* ≥ 0.05.

OL-FS13 improved the neuroethology of CI/R rats, but this effect was inhibited by agomiR (Fig. **4C**). Similarly, TTC staining showed (Figs. **4A** and **4B**) the miR-21-3p agomiR antagonized the therapeutic ability of OL-FS13 on cerebral infarct volume in CI/R rats.

**Fig. (5):**
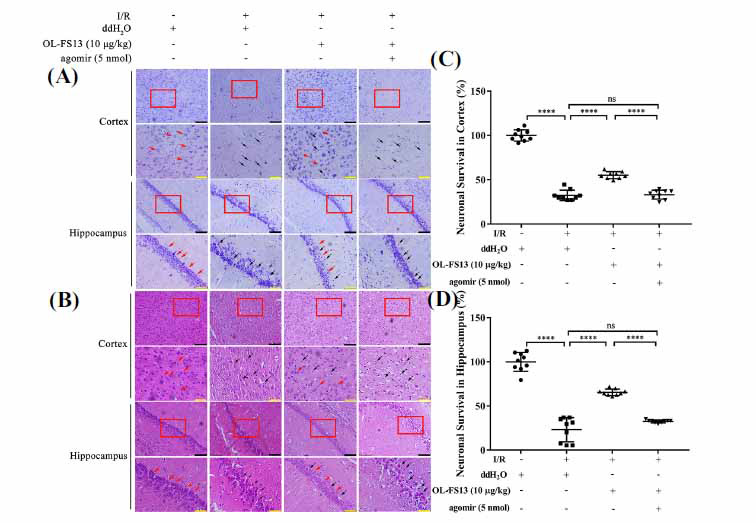
Overexpression of miR-21-3p interfered with the neuroprotection of OL-FS13 at the tissue level. (**A** and **B**) Nissl staining and H&E staining (red rectangle boxes indicated the enlarged observation areas; red arrows indicated normal cells; black arrows indicated abnormal individuals; black bar: 100 μm; yellow bar: 50 μm); (**C** and **D**) Bars showed the proportion of surviving neurons in hippocampal (**C**) and cortical (**D**) areas based on the results of Nissl staining. ****P < 0.0001,^ns^*P* ≥ 0.05.

Brain tissue morphology of each group was determined by H&E and Nissl staining. As Figs. (**5A** and **5B**) demonstrated, normal rats displayed intact cortical and hippocampal structures, with orderly cell arrangement and normal morphology. In contrast, CI/R rats showed abnormal cell morphology and arrangement, significantly alleviated by OL-FS13 treatment. Nissl staining of the hippocampus (Fig. **5C**) and cortex (Fig. **5D**) showed that agomiR intervention completely inhibited the ability of OL-FS13 to alleviate tissue injury (*P* < 0.0001, agomiR *vs*. OL-FS13; *P* ≥ 0.05, *vs*. I/R).

#### 3.3.3 Overexpression of miR-21-3p Inhibited Protective Ability of Peptide on CI/R Rats

**Fig. (6):**
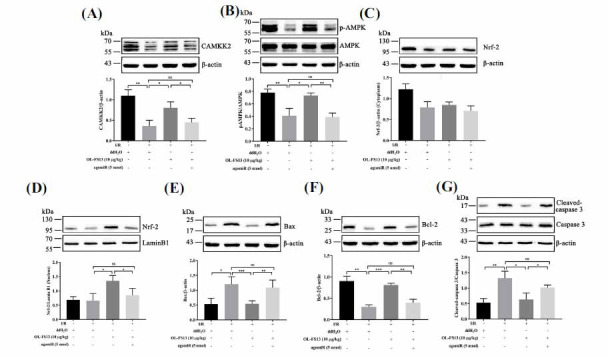
Overexpression of miR-21-3p inhibited the activation of CAMKK2/AMPK and Nrf-2 by OL-FS13. Control, I/R, I/R + OL-FS13, and I/R + OL-FS13 + agomiR were used for grouping. The expression levels of CAMKK2 (**A**), pAMPK/AMPK (**B**), Nrf-2 (**C** and **D**), Bax (**E**), Bcl-2 (F), Cleaved caspase 3/caspase 3 (G) (n=3) were evaluated. ***P < 0.001, **P < 0.01, *P < 0.05, ^ns^*P* ≥ 0.05.

As shown in Figs. (**6A** and **6B**), the CAMKK2/AMPK pathway was significantly inhibited by CI/R but was activated by OL-FS13 administration (*vs*. control), with these effects reversed by agomiR. Bax/Bcl-2/caspase 3 expression confirmed that the anti-apoptotic effects of OL-FS13 were significantly inhibited by miR-21-3p overexpression (Figs. **6E-G**). The miR-21-3p agomiR also inhibited the alleviation of OL-FS13 on Nrf-2 (Figs. **6C-D**). These results suggest the importance of miR-21-3p inhibition in the neuroprotective effects of OL-FS13.

### 3.4 Relationship between Nrf-2 and CAMKK2 in Neuroprotective Effects of OL-FS13

Previous research has confirmed that OL-FS13 can inhibit oxidative stress by activating Nrf-2, thereby easing neuronal apoptosis [25]. Thus, PC12 cells and rats were then pretreated with ML385 (inhibitor of Nrf-2) and STO-609 (inhibitor of CAMKK2), respectively.

**Fig. (7):**
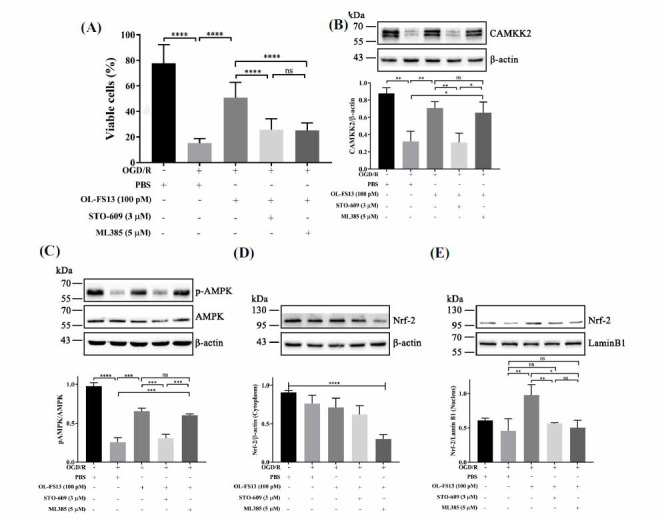
OL-FS13 activated Nrf-2 by increasing CAMKK2 expression. (**A**) The inhibitor of CAMKK2 (STO-609) and Nrf-2 (ML385) antagonized the cytoprotective effect of OL-FS13. (**B-E**) Control, OGD/R, OGD/R + OL-FS13, OGD/R + OL-FS13 + STO-609, and OGD/R + OL-FS13 + ML385 were used for grouping. The expression levels of CAMKK2 (**B**), pAMPK/AMPK (**C**), and Nrf-2 (**D** and **E**) (n=3) were evaluated. ****P < 0.0001, ***P < 0.001, **P < 0.01, *P < 0.05, ^ns^*P* ≥ 0.05.

**Fig. (8):**
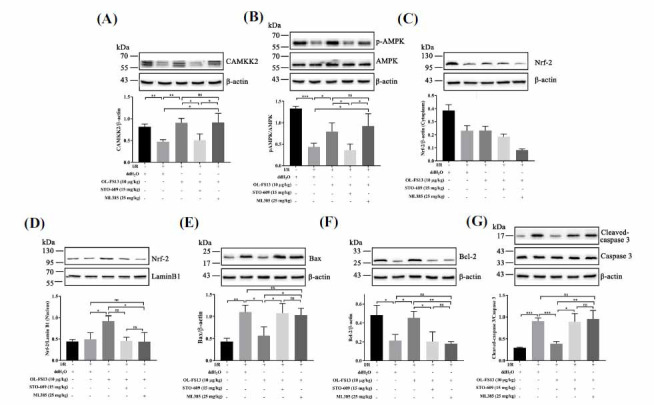
OL-FS13 attenuated CI/R injury by activating the CAMKK2/AMPK/Nrf-2 axis. Control, OGD/R, OGD/R + OL-FS13, OGD/R + OL-FS13 + STO-609, and OGD/R + OL-FS13 + ML385 were used for grouping. The expression levels of CAMKK2 (**A**), pAMPK/AMPK (**B**), Nrf-2 (**C** and **D**), Bax (**E**), Bcl-2 (**F**), Cleaved caspase 3/ caspase 3 (**G**) (n=3) were evaluated. ***P < 0.001, **P < 0.01, *P < 0.05, ^ns^*P* ≥ 0.05.

As shown in Fig. (**7A**), both ML385 and STO-609 inhibited the enhancing effects of OL-FS13 on cell viability. Figs. (**7B** and **7C**) showed that ML385 did not affect the expression of the CAMKK2/AMPK pathway under OL-FS13 intervention, whereas STO-609 inhibited the enhancing effects of OL-FS13 on Nrf-2 (Figs. **7D** and **7E**). Based on the Bax/ Bcl-2/caspase 3 results in Figs. (**8E-G**), both ML385 and STO-609 antagonized the anti-apoptotic effects of OL-FS13 in the CI/R rats. ML385 did not affect CAMKK2/AMPK expression in the rat brain tissue under OL-FS13 intervention (Figs. **8A** and **8B**), but STO-609 inhibited the enhancing effects of OL-FS13 on Nrf-2 (Figs. **8C** and **8D**).

The research showed that the activation of Nrf-2 by OL-FS13 occurs as a downstream event of CAMKK2/AMPK activation, thus indicating that OL-FS13 plays a neuroprotective role through the miR-21-3p/CAMKK2/AMPK/Nrf-2 axis.

## 4 DISCUSSION

Stroke causes long-term burdens on patients and society [49, 50]. Due to its multifaceted pathophysiology, current treatments for IS remain unsatisfactory. Early reperfusion is the accepted standard for IS treatment; however, outcomes are often difficult to predict due to the complex pathological changes after recanalization [49, 51]. Therefore, it’s important to explore the mechanisms of IS injury and related medicines to design effective treatments.

We previously identified OL-FS13 from *O. livida* [25] and explored the protective mechanism of OL-FS13 against OGD/R and CI/R injury in rats. Based on multi-omics sequencing, we determined the critical role of miR-21-3p in OL-FS13-mediated CI/R treatment. We found the role of miR-21-3p in OL-FS13 neuroprotection both *in vitro* and *in vivo* (apparent, tissue, and protein levels). Our results indicated that OL-FS13 alleviates OGD/R and CI/R injury by regulating the miR-21-3p/CAMKK2/AMPK/Nrf-2 axis.

As endogenously expressed in small noncoding RNAs, miRNAs are gene regulators controlling various pathophysiological signs of progress [21, 52]. In IS, miRNAs can not only serve to regulate the pathological mechanisms related to risk factors but can also serve as biomarkers to evaluate the severity of brain injury, suggesting potential advantages in CI/R research [53, 54]. Typically, miRNAs act by modulating mRNAs through “translational repression,” resulting in the impaired activity of their target genes [55]. These molecules are involved in various biological processes [24, 56].

In this research, we found that miR-21-3p targeted CAMKK2 (Fig. **1**). CAMKK2 is a serine/threonine protein kinase that regulates intracellular Ca^2+^ homeostasis and plays a key role in apoptosis [57, 58]. In CI/R, rapid depletion of ATP depolarizes the plasma membrane, which activates the voltage-dependent calcium channels in presynaptic and somatic mitochondria [3, 59]. Calcium overload leads to mitochondrial uncoupling, reduced ATP synthesis, and neuronal death [60, 61]. Considering the important role of calcium overload in CI/R injury, CAMKK2 with calcium-regulating function may be an effective drug target for CI/R therapy [62].

AMPK is a critical substrate involved in the dynamic regulation of Ca^2+^-ATP by CAMKK2 [63-65]. AMPK can trigger various signaling pathways conducive to ATP production [66-68]. In this research, OL-FS13 intervention significantly improved OGD/R- and CI/R-induced inhibition of the CAMKK2/AMPK signaling pathway (Fig. **2E-H**). The overexpression of miR-21-3p in PC12 cells also inhibited the protective effects of OL-FS13 *in vitro* and reversed its ability to activate the CAMKK2/AMPK pathway (Fig. **3**).

Similarly, miR-21-3p overexpression also inhibited the therapeutic effects of OL-FS13 on cerebral infarct volume (Figs. **4A** and **4B**), neurological function (Fig. **4C**), and tissue damage (Fig. **5**) in CI/R rats. CAMKK2 was found to play a crucial role in apoptosis [69]. As seen in Fig. (**6**), the overexpressed of miR-21-3p inhibited not only activation of the CAMKK2/AMPK pathway by OL-FS13 in CI/R rats but also antagonized the anti-apoptotic ability of OL-FS13 (based on Bax/Bcl-2/caspase 3 expression). The results above show that miR-21-3p is an important target of OL-FS13, and OL-FS13 exerts its neuroprotection *via* the miR-21-3p/CAMKK2/AMPK axis.

The brain is extremely sensitive to changes in ATP and vulnerable to oxidative stress [70]. In CI/R, calcium overload is also closely related to oxidative stress, resulting in a vicious cycle of continuous damage to tissues and cells, eventually leading to infarction and neurological dysfunction [60]. We previously showed that OL-FS13 inhibits CI/R-induced oxidative and endoplasmic reticulum stress by enhancing the Nrf-2/ARE signaling pathway [25]. In our study, MTS assay revealed that CAMKK2 and Nrf-2 were key proteins for the neuroprotective of OL-FS13 (Fig. **7A**). Interestingly, inhibition of Nrf-2 did not affect the activation of CAMKK2/AMPK by OL-FS13 (Figs. **7B-C**, **8A-B**). However, inhibition of CAMKK2 inhibited the enhancement of Nrf-2 expression by OL-FS13 (Figs. **7E** and **8D**). In other words, the neuroprotective effects of OL-FS13 involve the miR-21-3p/CAMKK2/AMPK/Nrf-2 axis, and its anti-oxidative ability occurs as a downstream event of Ca^2+^-ATP regulation.

Previous studies on miR-21-3p have primarily been about cancer and diabetes [71-74]. In addition, CAMKK2/AMPK research has primarily concentrated on cancer, while I/R research has mainly focused on myocardial I/R therapy [46, 65, 69, 75]. As such, we not only explored the possible role of miR-21-3p in the CI/R domain for the first time and provided support for its impact on neurological diseases but also provided further evidence for the important role of CAMKK2/AMPK in CI/R therapy. Of note, OL-FS13 may not only serve as a potential anti-CI/R molecule but also as an anchor point to explore the endogenous mechanisms of disease using exogenous peptides, providing additional opportunities to explore CI/R disease mechanisms.

## CONCLUSION

In conclusion, this study is the first to report on the possible role of miR-21-3p in CI/R. We also determined that the effects of OL-FS13 on CI/R and OGD/R may occur *via* the miR-21-3p/CAMKK2/AMPK/Nrf-2 axis, suggesting that miR-21-3p may be the target for its neuroprotective effects.

## Data Availability

The data supporting this study's findings are available from the corresponding author upon reasonable request.
