# Supplementary material for: OL-FS13 Alleviates Cerebral Ischemia-reperfusion Injury by Inhibiting miR-21-3p Expression
Source: Curr Neuropharmacol. 2023 Sep 25;21(12):2550–62. doi: 10.2174/1570159X21666230502111013 (PMC10616927; doi:10.2174/1570159X21666230502111013)
Supplement: Supplementary file 1 — Supplementary material is available on the publisher’s website along with the published article. [file CN-21-2550_SD1.pdf]

Supplementary Material

OL-FS13 Alleviates Cerebral Ischemia-reperfusion Injury by Inhibiting miR-21-3p Expression

Naixin Liu[1,#], Yan Fan[1,#], Yilin Li[1,#], Yingxuan Zhang[1], Jiayi Li[1], Yinglei Wang[1], Zhuo Wang[1], Yixiang Liu[2], Yuansheng Li[1], Zijian Kang[1], Ying Peng[1], Zeqiong Ru[1], Meifeng Yang[1,*], Chengan Feng[1,*], Ying Wang[2,*] and Xinwang Yang[1,*]

[1]Department of Anatomy and Histology & Embryology, Faculty of Basic Medical Science, Kunming Medical University, Kunming 650500, Yunnan, China; [2]Key Laboratory of Chemistry in Ethnic Medicinal Resources & Key Laboratory of Natural Products Synthetic Biology of Ethnic Medicinal Endophytes, State Ethnic Affairs Commission & Ministry of Education, School of Ethnic Medicine, Yunnan Minzu University, Kunming, Yunnan, 650504, China

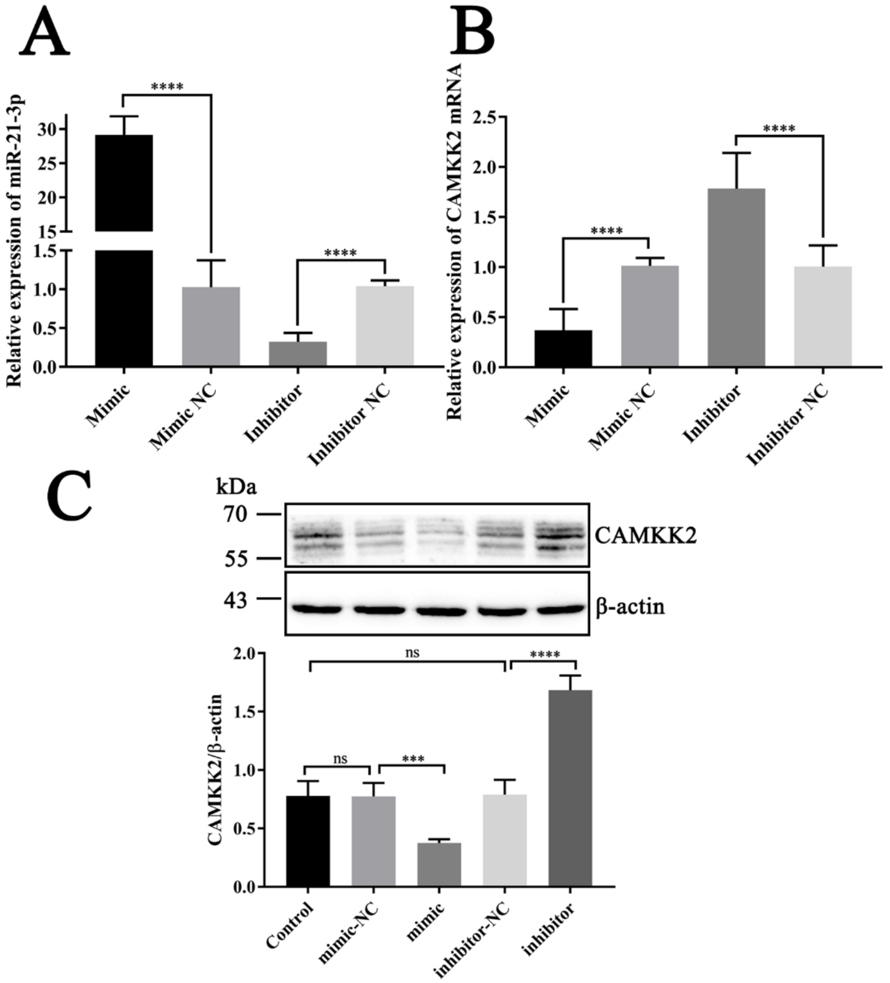

Supplementary Fig. (S1). Effects of miR-21-3p mimic and inhibitor on expression levels of miR-21-3p/CAMKK2 in PC12 cells. **A & B**. Mimics of miR-21-3p promoted miR-21-3p expression and inhibited CAMKK2 mRNA expression, while inhibitors inhibited the miR-21-3p expression and promoted CAMKK2 mRNA expression. **C**. Effects of mimics and inhibitors on CAMKK2 expression. $^{****}P < 0.0001$, $^{***}P < 0.001$, $^{ns}P \geq 0.05$.

1875-6190/23 $65.00+.00 © 2023 Bentham Science Publishers

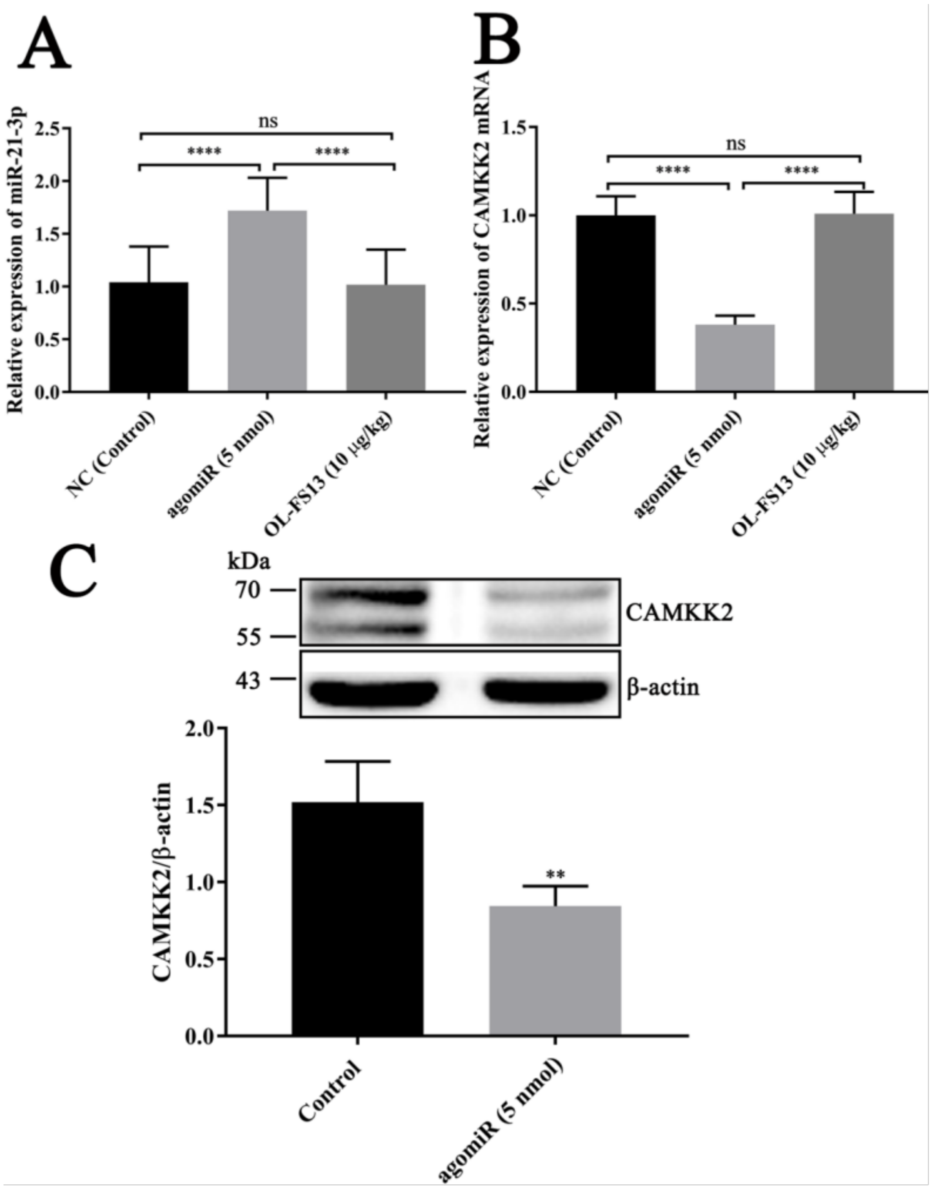

Supplementary Fig. (S2). Effects of miR-21-3p agomiR on miR-21-3p/CAMKK2 expression in the cerebral cortex of rats. **A & B.** AgomiR of miR-21-3p promoted miR-21-3p expression and inhibited CAMKK2 mRNA expression, while administration of OL-FS13 (without I/R surgery) did not affect miR-21-3p or CAMKK2 mRNA expression in the cerebral cortex of rats. **C.** Administration of miR-21-3p agomiR inhibited CAMKK2 expression in brain tissue. $^{****}P < 0.0001$, $^{**}P < 0.01$, $^{ns}P \geq 0.05$.
